# Integrated molecular pathway analysis informs a synergistic combination therapy targeting PTEN/PI3K and EGFR pathways for basal-like breast cancer

**DOI:** 10.1186/s12885-016-2609-2

**Published:** 2016-08-02

**Authors:** Qing-Bai She, Sofia K. Gruvberger-Saal, Matthew Maurer, Yilun Chen, Mervi Jumppanen, Tao Su, Meaghan Dendy, Ying-Ka Ingar Lau, Lorenzo Memeo, Hugo M. Horlings, Marc J. van de Vijver, Jorma Isola, Hanina Hibshoosh, Neal Rosen, Ramon Parsons, Lao H. Saal

**Affiliations:** 1 Program in Molecular Pharmacology and Chemistry and Department of Medicine, Memorial Sloan-Kettering Cancer Center, New York, NY USA; 2 Markey Cancer Center, University of Kentucky College of Medicine, Lexington, KY USA; 3 Department of Pharmacology and Nutritional Sciences, University of Kentucky College of Medicine, Lexington, KY USA; 4 Division of Oncology and Pathology, Clinical Sciences, Lund University, Lund, Sweden; 5 Institute for Cancer Genetics, Columbia University Medical Center, New York, NY USA; 6 Herbert Irving Comprehensive Cancer Center, Columbia University, New York, NY USA; 7 Department of Medicine, Columbia University, New York, NY USA; 8 Department of Pathology, Seinäjoki Central Hospital, Seinäjoki, Finland; 9 Department of Experimental Oncology, Mediterranean Institute of Oncology, Catania, Italy; 10 Department of Pathology, Netherlands Cancer Institute, Amsterdam, The Netherlands; 11 Department of Pathology, Academic Medical Center, Amsterdam, The Netherlands; 12 Institute of Medical Technology, University of Tampere, Tampere, Finland; 13 Department of Pathology, Columbia University, New York, NY USA; 14 Department of Oncological Sciences and The Tisch Cancer Institute, Icahn School of Medicine at Mount Sinai, New York, NY USA; 15 Translational Oncogenomics Unit, Division of Oncology and Pathology, Lund University Cancer Center, Medicon Village 404-B2, SE-22381 Lund, Sweden

**Keywords:** Basal-like, Breast cancer, EGFR, PTEN, Combination therapy

## Abstract

**Background:**

The basal-like breast cancer (BLBC) subtype is characterized by positive staining for basal mammary epithelial cytokeratin markers, lack of hormone receptor and HER2 expression, and poor prognosis with currently no approved molecularly-targeted therapies. The oncogenic signaling pathways driving basal-like tumorigenesis are not fully elucidated.

**Methods:**

One hundred sixteen unselected breast tumors were subjected to integrated analysis of phosphoinositide 3-kinase (PI3K) pathway related molecular aberrations by immunohistochemistry, mutation analysis, and gene expression profiling. Incidence and relationships between molecular biomarkers were characterized. Findings for select biomarkers were validated in an independent series. Synergistic cell killing in vitro and in vivo tumor therapy was investigated in breast cancer cell lines and mouse xenograft models, respectively.

**Results:**

Sixty-four % of cases had an oncogenic alteration to *PIK3CA*, PTEN, or *INPP4B*; when including upstream kinases HER2 and EGFR, 75 % of cases had one or more aberration including 97 % of estrogen receptor (ER)-negative tumors. PTEN-loss was significantly associated to stathmin and EGFR overexpression, positivity for the BLBC markers cytokeratin 5/14, and the BLBC molecular subtype by gene expression profiling, informing a potential therapeutic combination targeting these pathways in BLBC. Combination treatment of BLBC cell lines with the EGFR-inhibitor gefitinib plus the PI3K pathway inhibitor LY294002 was synergistic, and correspondingly, in an in vivo BLBC xenograft mouse model, gefitinib plus PI3K-inhibitor PWT-458 was more effective than either monotherapy and caused tumor regression.

**Conclusions:**

Our study emphasizes the importance of PI3K/PTEN pathway activity in ER-negative and basal-like breast cancer and supports the future clinical evaluation of combining EGFR and PI3K pathway inhibitors for the treatment of BLBC.

**Electronic supplementary material:**

The online version of this article (doi:10.1186/s12885-016-2609-2) contains supplementary material, which is available to authorized users.

## Background

Breast cancer (BC) is comprised of several molecular subtypes with varied biological and clinical characteristics. Of these, the four most discernible subtypes by gene expression profiling include the two estrogen-receptor (ER)-positive subtypes, luminal A and B which are characterized by low and high proliferation, respectively; the HER2 subtype characterized by overexpression of genes in the HER2 (*ERBB2*) amplicon; and the basal-like BC (BLBC) subtype characterized by expression of basally-oriented mammary epithelial cell markers such as cytokeratins 5 and 14 (CK5/14), lack of hormone receptors and HER2 expression (“triple-negative”), and high genomic instability [1]. Although therapies that target estrogen pathways or the HER2 receptor are part of the standard armamentarium in clinical use today, currently no specific drugs that target the basal-like subtype are approved. The basal-like subtype also has a particularly poor prognosis, underscoring the need for improved therapeutic options for women with this class of cancer.

Recent studies point to the importance of the phosphatidylinositol 3-kinase (PI3K) and phosphatase and tensin homolog deleted on chromosome ten (PTEN) oncogene/tumor suppressor axis in breast tumorigenesis. The *PIK3CA* oncogene, which encodes the PI3K p110 catalytic subunit alpha and phosphorylates phosphatidylinositol 3,4-bisphosphate (PIP2) to PIP3, has been shown to have activating mutations in approximately 30 % of BCs, primarily within ER-positive cases [2–6]. Although the tumor suppressor PTEN, a lipid phosphatase acting in direct enzymatic opposition to PI3K, is infrequently inactivated by mutations in sporadic BC (5 %), PTEN protein expression is significantly reduced in ~25 % breast tumors, more commonly in ER-negative cancer and in particular within BLBC, and rarely coinciding with *PIK3CA* mutation [2, 7]. *PTEN* is also frequently grossly mutated in *BRCA1*-hereditary BC, a group of tumors that usually exhibit the basal-like phenotype [7, 8], and new nuclear roles for PTEN in maintaining genome stability have been identified. Another lipid phosphatase, inositol polyphosphate 4-phosphatase type II (INPP4B), has recently been shown to have potential tumor suppressor activity [9, 10]. The tumor suppressive role of INPP4B is linked to its function in dephosphorylating PIP2, depleting this substrate of PI3K, and accordingly loss of INPP4B leads to increased PIP3-mediated signaling to AKT and is associated with poor outcome (reviewed in [11]). Additionally, numerous upstream receptor tyrosine kinases (RTKs), such as HER2 and EGFR, have been shown to activate PI3K pathway signaling [12, 13], and EGFR also potently signals through the RAS/RAF/MEK (MAPK) pathway [14]. Constitutive activation of PI3K pathway by *PTEN* mutation/loss or *PIK3CA* mutation could render tumor cells independent of RTKs for malignant transformation and maintenance, which leads to resistance to HER2-targeted therapies in BC and EGFR-targeted therapies in glioma [15–20].

To understand the natural history of PI3K pathway activating alterations and relate them to BC subtypes and other molecular and genetic alterations, we have performed an extensive analysis of pathway biomarker lesions in an unselected cohort of breast tumors. We examined the incidence of pathway lesions, and discovered novel associations to standard clinicopathological markers and to BC subtypes including the frequent coincident loss of PTEN and overexpression of EGFR in BLBC. We then assessed whether a combination therapeutic strategy targeting both the PTEN/PI3K pathway and EGFR would be more effective than monotherapy using in vitro and in vivo models.

## Methods

### Human breast cancer samples

For 116 primary BC patients consented and treated at Columbia University Herbert Irving Comprehensive Cancer Center/New York-Presbyterian Hospital, formalin-fixed paraffin-embedded (FFPE) tumor blocks and DNA and total RNA isolated from corresponding frozen tumor specimens were obtained (Columbia cohort; Table 1). All samples were blinded and anonymized and obtained with ethics approval from Columbia University’s Institutional Review Board. This unselected cohort is comprised of patients diagnosed between 1986 and 2003 with all stages of BC and the patients received standard of care therapies. FFPE tissue microarrays for an independent series of 295 consecutive women with stage I and II breast cancer treated at the Netherlands Cancer Institute (NKI) was obtained [21, 22].

**Table 1:** PTEN/PI3K biomarkers in 116 unselected breast cancers

Variable	*n*	%	Total
Breast tumors	116	100.0 %	116
Median age (range)	53 years	(30–89)	
ER+	82	70.7 %	116
ER–	34	29.3 %	
PgR+	71	61.2 %	116
PgR–	45	38.8 %	
Size <20	36	31.3 %	115
20–49	64	55.7 %	
50+	15	13.0 %	
Grade 1	8	7.0 %	115
Grade 2	32	27.8 %	
Grade 3	75	65.2 %	
Node+	56	53.3 %	105
Node–	49	46.7 %	
HER2+	23	20.4 %	113
HER2–	90	79.6 %	
Ki67 IHC+	35	38.0 %	92
Ki67 IHC–	57	62.0 %	
S–phase ≤6 %	31	33.3 %	93
S–phase >6 %,<10 %	17	18.3 %	
S–phase ≥10 %	45	48.4 %	
CK5/14 IHC+	19	17.4 %	109
CK5/14 IHC–	90	82.6 %	
p53 mut	44	38.3 %	115
p53 wt	71	61.7 %	
EGFR IHC+	27	24.1 %	112
EGFR IHC–	85	75.9 %	
PTEN abrogated (PTEN IHC- or mut)	26	24.3 %	107
PTEN norm	81	75.7 %	
PTEN IHC–	25	23.4 %	107
PTEN IHC+	82	76.6 %	
PTEN mut	4	3.6 %	112
PTEN wt	108	96.4 %	
PIK3CA mut	29	25.0 %	116
PIK3CA wt	87	75.0 %	
INPP4B mRNA low	20	21.3 %	94
INPP4B mRNA norm	74	78.7 %	
Any PTEN/PI3K/INPP4B	64	64.0 %	100
None PTEN/PI3K/INPP4B	36	36.0 %	
Any PTEN/PI3K/INPP4B/EGFR/HER2	80	75.5 %	106
None PTEN/PI3K/INPP4B/EGFR/HER2	26	24.5 %	

### Tissue microarray construction

Tissue microarrays (TMAs) were constructed for the Columbia cohort by the Experimental Molecular Pathology Core Facility of the Herbert Irving Comprehensive Cancer Center utilizing a Manual Tissue Arrayer-1 device (Beecher Instruments, Sun Prairie, WI). Tumor and normal tissue areas were identified using hematoxylin and eosin-stained sections, with 3 representative tumor and 3 representative normal tissue cores of 1-mm diameter taken from each FFPE case and inserted into the recipient blocks.

### Immunohistochemistry and Western blotting

The Columbia cohort were analyzed by immunohistochemistry (IHC) for expression of the following proteins: PTEN, EGFR, cytokeratins 5 and 14 (CK5/14), stathmin, and Ki67. The NKI series TMAs were analyzed herein for CK5/14 and stathmin by IHC and previously immunostained for PTEN protein [23]. PTEN IHC was performed on 4-μm FFPE whole-mount tissue sections using the monoclonal 138G6 PTEN antibody (Cell Signaling, Danvers, MA) at 1:200 dilution for 2 h at room temperature. Microwave antigen retrieval was accomplished using the Dako pH 9 solution for 20 min, followed by automated staining using the DakoCytomation TechMate 500 staining system with manufacturer’s recommended reagents and Dako EnVision + signal detection (Dako, Carpinteria, CA). PTEN staining intensity scores for invasive tumor and non-neoplastic cells was evaluated and the tumors classified as PTEN-negative (PTEN^−^) and PTEN-positive (PTEN^+^) as described previously [2]. Normal epithelial and endothelial cell staining were used as internal positive controls. EGFR IHC was performed using antibody 31G7 (Zymed/Invitrogen, South San Francisco, CA) at 375 ng/ml (1:40) on 4-μm TMA sections. Slides were treated for 5 min with Dako proteinase K and washed prior to incubating in primary antibody for 45 min at room temperature. Anti-mouse secondary antibody was applied for 30 min, and the signal detected using diaminobenzidine (DAB) chromogen for 3 min followed by DAB Enhancer for 4 min (all Dako). The slides were counterstained with Gils Hematoxylin. EGFR staining was evaluated using a threshold of ≤10 % positive tumor cells as EGFR-negative (EGFR^−^) and >10 % positive tumor cells as EGFR-positive (EGFR^+^). CK5/14 IHC was performed on TMAs using an antibody cocktail and the cases scored CK5/14-positive (CK5/14^+^) or CK5/14-negative (CK5/14^−^) as described previously [24]. Stathmin IHC was performed on TMAs and evaluated as previously described [25]. Ki67 mouse monoclonal antibody Ki-S5 (Dako) was used at 1:50 on whole-mount tissue sections. IHC was performed on a Dako autostainer, using a Vector biotinylated secondary anti-mouse antibody (1:200 for 30 min) and Vectastain Elite detection with DAB (Vector Laboratories, Burlingame, CA). Sections were counterstained with methyl green (Sigma, St. Louis, MO). Appropriate positive and negative (staining lacking primary antibodies) controls were used in each batch of staining. Evaluation of Ki67 was performed by determining the percentage of positive tumor nuclei as evaluated by the CASS 200 Image Analyzer (Becton Dickinson, San Jose, CA). Cases were considered Ki67-positive when ≥20 % of the tumor cells showed evidence of nuclear expression. For Western blotting, the following antibodies were used: pEGFR (Tyr1068; #3777), pAKT (Ser473; #9271), AKT (#9272) (all Cell Signaling), EGFR (Santa Cruz Biotechnology, Santa Cruz, CA; sc-03), and β-actin (Sigma-Aldrich, St. Louis, MO; AC-74 #A5316).

### HER2 amplification

*HER2* amplification was assessed for 101 Columbia cases using chromogenic in situ hybridization (CISH) on TMAs, with six or more signals per cell in >50 % of cancer cells scored as *HER2*-amplified (HER2^+^) [26]. Tumors in which the majority of cells contained 5 or fewer signals per nucleus were scored *HER2*-non-amplified (HER2^−^). HER2 scores by IHC were used for 6 additional cases for which CISH hybridization failed; HER2 IHC methods are described in [2]. The clinical diagnostic pathology evaluation for HER2 was utilized for another 6 cases lacking *HER2* CISH and IHC data.

### PCR and sequence analysis

Sequencing of *PIK3CA* exons 1, 2, 4, 5, 7, 9, 12, 13, 18, and 20 for the Columbia cohort of cases has been described previously [2]. In the present study we have performed additional mutational analysis of the Columbia cohort for *PTEN* and *TP53*. Mutational screening of *TP53* exons 2 through 11 was performed using 8 pre-validated primer assays and direct bi-directional sequencing (Agencourt Bioscience, Beverly, MA). *PTEN* and *TP53* sequence traces were analyzed using Mutation Surveyor (Softgenetics, State College, PA) and Polyphred (http://droog.gs.washington.edu/polyphred/), respectively.

### Gene expression profiling

Gene expression profiles were generated for 95 Columiba cases using Agilent 44 K microarrays following the manufacturer protocol and scanned on an Agilent Microarray Scanner. Stratagene Universal Human Reference RNA was used as the common control sample. Images were analyzed using the Agilent Feature Extraction Software and the raw data loaded into BASE [27] for processing. Background-corrected intensities (mean foreground – median background) were filtered for values with A > 0.5 (A = log_10_(channel1 * channel2)/2) and the data normalized using the Lowess algorithm. Tumors were dichotomized into *INPP4* low and high categories, with low defined as the log2 ratio <0 (20 cases) in expression as measured by the microarray *INPP4* probe A_24_P915492. The tumors were classified into the Hu et al. [28] intrinsic molecular subtypes (luminal A, luminal B, HER2, basal-like, or normal-like): 301 unique gene symbols from Hu et al. mapped to 273 gene symbols in our data. The log2 data were averaged on gene symbol and centered across all samples, and classified into subtypes based on the best Pearson correlation to the Hu centroids. The tumors were classified by the PTEN-loss signature [25]: 173 unique gene symbols from Saal et al. mapped to 143 gene symbols in the 44 K microarray data. The data were similarly averaged and centered as above, and the PTEN-loss-signature present or absent score calculated as described previously with a sample |score| <0.2 set as unclassified [25]. Microarray data are available from the NCBI Gene Expression Omnibus (http://www.ncbi.nlm.nih.gov/geo/) under accession GSE74667. Previously published microarray data and molecular subtyping was retrieved for the NKI series [21, 22].

### Cell viability and apoptosis assays

Cells were seeded in 96-well plates at a density of 5,000-8,000 cells in triplicates. After 24 h, cells were treated with different concentrations of the indicated inhibitors and incubated at 37 °C. The cells were cultured for 4 days and then the number of viable cells was measured by CellTiter-Glo luminescent cell viability assay according to the manufacturer’s standard protocol (Promega). Analysis for synergistic drug combination efficacy was carried out using the method of Chou and Talalay [29] using CompuSyn 3.0.1 (ComboSyn Inc., Paramus, NJ). Combination index (CI) values of <1 are taken to indicate synergistic interaction between drugs, and CI values of >1 indicate drug antagonism. To measure apoptosis, both adherent and floating cells were harvested after drug treatment, and the cell nuclei were stained with ethidium bromide [30]. Detection and quantitation of apoptotic cells (sub-G1 fraction) were performed by flow cytometric analysis as described previously [18].

### Animal studies

Six-week-old *nu/nu* athymic female mice (NCI-Frederick Cancer Center, Frederick, MD) were maintained in pressurized ventilated cages. Experiments were carried out under an IACUC approved protocol and institutional guidelines for the proper and humane use of animals in research were followed. MDA-MB-468 xenograft tumors were generated by transplanting 1.0–1.5 × 10^7^ MDA-468 cells in a 1:1 mixture of media and Matrigel (BD Biosciences, San Jose, CA) into the right flank (200 μl/mouse). After 7–10 days, the mice bearing tumors 6–7 mm in diameter were randomized among control and the various treated groups (5 mice/group). Gefitinib prepared as a lactate salt (pH 5.2) was administrated orally at a dose of 150 mg/kg/day × 5 consecutive days each week for 3 weeks. PWT-458 was freshly prepared in PBS, and administrated by intravenous injection at a dose of 100 mg/kg/day × 5 consecutive days each week for 3 weeks. For the combination treatment, PWT-458 was administrated 3–4 h before gefitinib treatment. The average tumor diameter (two perpendicular axes of the tumor were measured) was measured in control and treated groups using a caliper. The data are expressed as the increase or decrease in tumor volume in mm^3^ (volume = π/6 × [larger diameter] × [smaller diameter]^2^). For Western blot experiments, animals with established tumors were sacrificed 5 h after treatment and tumor lysates were prepared in 2 % SDS buffer as described previously [18].

### Data visualization and statistical analysis

Hierarchical clustering of marker data and tumors were performed in Cluster 3.0 [31] utilizing the Pearson correlation distance metric (centered) and complete linkage algorithm and the data visualized using Java Treeview [32]. The Pearson χ^2^ test was used for correlation analyses between two binary variables or between variables with more than 2 unordered groups, and the χ^2^ test for trend was used for analyses between variables with more than 2 ordered groups. Calculations were performed using R version 3.1.0. All tests were two-sided and a *P*-value < 0.05 was used as the cut-off for decisions of statistical significance.

## Results

### Distribution of biomarker lesions

Our unselected Columbia cohort of 116 BCs is representative of the population seen at the Herbert Irving Comprehensive Cancer Center (Table 1). The median age at diagnosis was 53 (range 30 to 89), 71 % of cases were ER-positive, 61 % progesterone receptor (PgR) positive, 31 % were <2-cm and 87 % were <5-cm. The majority of cases were of higher grade, approximately half had S-phase fraction >10 %, and 53 % had lymph node-positive disease at diagnosis. Biomarker status was determined for PTEN (mutations and immunohistochemistry [IHC]), *PIK3CA* (mutations), *HER2* (amplification), EGFR (IHC), *INPP4B* (mRNA expression), CK5/14 (IHC), and *TP53* (mutations). Thirty-eight % of cases had deleterious mutations of *TP53*, in line with the expected *TP53* mutation rate in BC [33]. Seventeen % were positive for the basal cytokeratins CK5/14 (which herein we use to define immunohistochemical BLBC; IHC-BLBC), consistent with reported rates [34]. As shown in Table 1, alteration of pathway members PTEN (24 %), *PIK3CA* (25 %), *HER2* (20 %), EGFR (24 %), and *INPP4B* (21 %) was common and the rates were in accordance with the literature [2, 35–40]. When these five markers were combined into a ‘pathway hit’ variable (positive if any of the 5 markers were altered), 75 % of breast tumors harbored one or more alterations that contribute to PTEN/PI3K pathway activation.

### PTEN/PI3K pathway-related tumor clusters

**Fig. 1:**
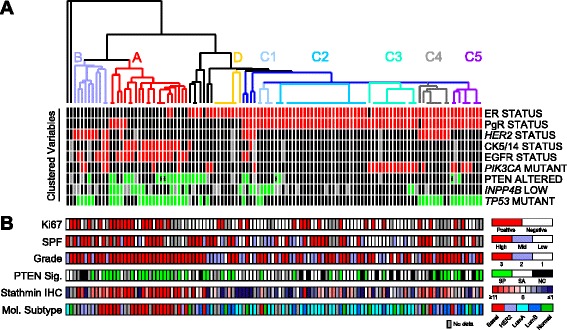
Clustering of unselected breast tumors. **a** The biomarker variables ER, PgR, PIK3CA, TP53, HER2, PTEN, EGFR, and CK5/14 were used to hierarchically cluster 116 breast tumors. Biomarker data are colored such that red = positive, black = negative, green = mutant (TP53) or lost (INPP4B, PTEN; asterix denotes PTEN mutation), and grey = missing data. **b** Below the heatmap are additional clinicopathological annotations not used to generate the clustered heatmap: Ki67, S-phase fraction (SPF), histological grade, PTEN-loss signature status, and intrinsic molecular subtype by gene expression profilling. For each annotation, color key is shown to the right (grey = missing data). SP = signature present; SA = signature absent; NC = not classified

To visualize these biomarker data we utilized unsupervised hierarchical clustering. As shown in Fig. 1a, the 116 tumors were clustered into four clusters by virtue of the intrinsic relationships between the markers ER, PgR, PTEN, *PIK3CA*, *HER2*, EGFR, *INPP4B*, CK5/14, and *TP53*. Cluster A, containing 22 tumors, is largely defined by lack of ER and/or PgR hormone receptor expression (15/22; 68 % expressed neither receptor and no case co-expressed both receptors), EGFR-overexpression (18/22; 82 %), IHC-BLBC status (15/22; 68 %), mutated *TP53* (18/22; 82 %), and PTEN-loss (14/21; 67 %). When annotated to additional clinicopathological data not utilized in generating the clusters (Fig. 1b), we note that this cluster contained entirely histological grade 3 tumors (22/22; 100 %) and had the highest proportion of Ki67-positive tumors (13/17; 76 %). Ten tumors formed cluster B, which is characterized primarily by lack of hormone receptor expression (0 %), *HER2*-amplification (80 %), and high histological grade (70 %). A large group of 67 tumors form cluster C, which are characterized by being predominantly ER-postive and PgR-postive (65/67; 97 %). Cluster C subclusters are defined by loss of *INPP4B* (subcluster C1), general lack of any other aberrations (C2), *PIK3CA* mutation-positive without *TP53* mutation (C3) or with *TP53* mutation (C5), and *HER2*-amplified cases with *TP53* mutation (C4). Cluster D is defined by ER-postive/PgR-negative without other aberrations (Fig. 1a).

Microarray gene expression profiling (GEX) was performed to further discern biological insight into the biomarker-defined clusters. Cluster A tumors were predominantly classified as belonging to the basal-like intrinsic molecular subtype (termed GEX-BLBC herein) (16/18; 89 %), and 75 % of the tumors (15/18) were classified, using our previously described signature, as having a gene expression pattern of PTEN-loss (Fig. 1b). In line with expectations, 50 % of cluster B belonged to the HER2-enriched subtype by GEX, and 50 % of cluster B expressed the PTEN-loss-like signature. Clusters C and D were primarily luminal subtype by GEX (75 %: 51 % luminal A, 24 % luminal B) and only 29 % had an expression profile of PTEN-loss. Subcluster C4 (HER2-amplified) was predominantly HER2-enriched or luminal B subtype (75 %). As previously described [41, 42], the subgroups for BLBC status defined by GEX, CK5/14, or triple-negative receptors were not completely synonymous (Fig. 1). Finally, across the dataset the PTEN IHC-negative cases were predominantly classified as having the expression signature of PTEN-loss (*P* = 0.019).

The differences in the distribution of Ki67 status, tumor grade, intrinsic molecular subtypes, and presence of the PTEN-loss signature across the main tumor clusters A-D were significant (*P* = 0.0009, *P* = 0.0067, *P* < 0.0001, *P* = 0.0012, respectively). However, the distribution of lymph node status and S-phase fraction were not related to the biomarker clusters (*P* = 0.2947 and *P* = 0.2604, respectively) (Fig. 1b). We performed IHC for stathmin, a PTEN signature gene that we have previously shown to be upregulated in breast tumors with PI3K/PTEN pathway activation [25]. Confirming our previous findings, stathmin protein levels were significantly higher in tumors with PTEN protein loss (*P* = 0.0015), with PTEN abrogation (*P* = 0.0015), and in tumors expressing the PTEN-loss gene expression signature (*P* = 0.0005) (Fig. 1b).

### Associations between pathway lesions

We next wanted to evaluate the relationship of PTEN/PI3K/INPP4B/RTK pathway lesions to each other. We have previously reported in a series of Swedish stage II sporadic breast carcinomas that mutations of *PIK3CA* and loss of PTEN are mutually exclusive [2]. Confirming this in our independent unselected patient cohort, *PIK3CA* and PTEN loss were essentially mutually exclusive with only one tumor being both *PIK3CA* mutated and PTEN IHC-negative (*P* = 0.0116; Table 2). When including *PTEN* mutational data only two cases had mutant *PIK3CA* and abrogated PTEN (either PTEN IHC-negative or PTEN-mutant) (*P* = 0.0718). Together, this implies that there is little selective pressure to activate the pathway by hitting both enzymes at the PIP_3_ axis in breast tumors. Interestingly, the single case mutated for both *PTEN* and *PIK3CA* was PTEN IHC-positive and harbored a *PTEN* I28T mutation together with the *PIK3CA* E545K mutation. *PTEN* I28T mutation, to our knowledge, has not been reported in the literature in any tumor type and mutation at residue 28 has been reported in only three times (COSMIC Database, http://www.sanger.ac.uk/genetics/CGP/cosmic/). Given that this *PTEN* mutant was the only mutant (out of 4) that was PTEN IHC-positive, the functional relevance of this mutation is not clear. Intriguingly, more than half of the cases with loss or abrogation of PTEN protein expression overexpressed EGFR (*P* < 0.0001 and *P* < 0.0001, respectively), suggesting cooperation between EGFR and PTEN/PI3K signaling. Similarly, tumors with EGFR overexpression frequently had low *INPP4B* expression (*P* = 0.0189). No other significant associations were noted between PTEN/PI3K pathway alterations (Table 2).

**Table 2:** Associations between pathway alterations

	PTEN mut	PTEN wt	n	p	HER2+	HER2-	n	p	EGFR+	EGFR-	n	p	PIK3CA mut	PIK3CA wt	n	p	INPP4B low	INPP4B high	n	p
PTEN IHC-	3	20	103	**0.010**	2	23	107	0.117	14	11	107	**<0.0001**	1	24	107	**0.012**	6	11	85	0.111
PTEN IHC+	1	79			18	64			12	70			23	59			12	56		
PTEN abrogation					2	24	107	0.098	14	12	107	**<0.0001**	2	24	107	0.072	6	12	85	0.155
PTEN normal					18	63			12	69			22	59			12	55		
HER2+									8	14	110	0.150	6	17	113	0.871	3	15	91	0.624
HER2-									19	69			22	68			16	57		
EGFR+													5	22	112	0.436	8	12	90	**0.019**
EGFR-													22	63			11	59		
PIK3CA mut																	4	24	94	0.281
PIK3CA wt																	16	50		

Footnote: Significant *p-*values are indicated by bolding

Abbreviations: *mut* mutant, *wt* wild-type

### Pathway lesions and clinicopathological variables

Given the inherent biological groups identified by unsupervised hierarchical clustering analysis (Fig. 1), we queried the correlation of pathway lesions to common clinicopathological markers in BC (Table 3). Supporting prior reports, PTEN protein loss by IHC and the PTEN abrogated state (either PTEN IHC-negative or *PTEN*-mutated) was significantly more common in ER-negative tumors (*P* = 0.0007 and *P* = 0.0013, respectively) and PgR-negative tumors (*P* = 0.0003 and *P* = 0.0007). PTEN loss or abrogation was also significantly correlated to higher grade (*P* = 0.0017 and *P* = 0.0012, respectively) and larger tumor size (*P* = 0.0018 and *P* = 0.0005). Similarly, EGFR overexpression was associated to hormone receptor negativity (*P* < 0.0001) and increasing tumor grade (*P* = 0.0008), but not tumor size (*P* = 0.2160). Seventy-six % of *PIK3CA* mutants were ER-positive, and although larger studies have found an association between *PIK3CA* mutation and ER status [2, 6], this was not a significant enrichment in the present cohort perhaps as a consequence of limited sample size. Interestingly, *PIK3CA* mutations correlated with lower tumor grade (*P* = 0.0265) and lower S-phase fraction (*P* = 0.0416) (Table 3). This result was supported also by reduced Ki67 (*P* = 0.0121). Using the 5-biomarker ‘pathway hit’ variable, 97 % (33/34) of ER-negative tumors had one or more pathway alterations compared to 65 % of ER-positive tumors (*P* = 0.0004; Table 3). Similarly, 88 % of PgR-negative tumors had one or more pathway alterations compared to 67 % of PgR-positives (*P* = 0.0144; Table 3).

**Table 3:** Correlation of PI3K Pathway Lesions to Other Clinicopathological Markers

	ER+	ER–	*n*	*P*	PgR+	PgR –	*n*	*P*	p53 mut	p53 wt	*n*	*P*	CK5/14 +	CK5/14 –	*n*	*P*	Grade 1	Grade 2	Grade 3	*n*	*P*	<20 mm	20–49 mm	50 mm+	*n*	*P*	Node+	Node –	*n*	*P*	S–phase ≤6 %	S–phase 6-10 %	S–phase ≥10 %	*n*	*P*	Basal-like	HER2	Normal-like	LumA	LumB	*n*	*P*
PTEN IHC–	11	14	107	**0.0007**	8	17	107	**0.0003**	15	9	106	**0.006**	11	13	106	**<0.0001**	0	2	23	106	**0.002**	3	14	8	107	**0.002**	14	8	97	0.392	4	1	13	85	0.062	11	2	2	0	2	86	**0.0003**
PTEN IHC+	65	17			59	23			26	56			7	75			8	27	46			28	47	7			40	35			24	15	28			11	6	8	31	13		
PTEN mut	2	2	112	0.334	2	2	112	0.600	3	1	112	0.115	2	2	105	0.061	0	0	4	111	0.166	0	3	1	111	0.175	2	2	101	0.855	1	0	2	91	0.708	3	0	0	0	0	94	**0.048**
PTEN wt	78	30			68	40			39	69			15	86			8	31	68			35	58	14			53	44			30	16	42			20	10	10	34	17		
PTEN abrogated	12	14	107	**0.001**	9	17	107	**0.0007**	16	9	106	**0.003**	11	14	106	**<0.0001**	0	2	24	106	**0.0012**	3	14	9	107	**0.0005**	15	8	97	0.291	5	1	13	85	0.135	12	2	2	0	2	86	**<0.0001**
PTEN normal	64	17			58	23			25	56			7	74			8	27	45			28	47	6			39	35			23	15	28			10	6	8	31	13		
HER2+	13	10	113	0.092	13	10	113	0.548	13	10	112	0.058	3	18	108	0.740	1	7	15	112	0.806	7	13	3	113	0.979	13	8	103	0.437	3	3	10	90	0.177	3	7	3	2	3	92	**0.0003**
HER2–	67	23			57	33			31	58			15	72			7	24	58			28	50	12			43	39			26	14	34			19	3	8	31	13		
EGFR+	4	23	112	**<0.0001**	5	22	112	**<0.0001**	19	8	111	**<0.0001**	15	11	109	**<0.0001**	0	2	25	111	**0.0008**	6	16	5	112	0.216	13	9	102	0.583	5	3	14	89	0.112	16	3	1	0	0	91	**<0.0001**
EGFR–	74	11			63	22			24	60			4	79			8	29	47			28	47	10			42	38			25	13	29			7	6	10	32	16		
PIK3CA mut	22	7	116	0.480	21	8	116	0.153	11	18	115	0.966	4	22	109	0.753	4	10	14	115	**0.027**	10	16	3	115	0.568	16	11	105	0.474	13	3	9	93	**0.042**	5	3	5	12	3	95	0.446
PIK3CA wt	60	27			50	37			33	53			15	68			4	22	61			26	48	12			40	38			18	14	36			18	7	6	22	14		
INPP4B low	10	10	94	**0.012**	12	8	94	0.773	10	10	93	0.292	6	13	88	0.057	3	3	14	94	0.954	8	7	5	93	0.928	9	10	85	0.750	5	6	3	73	0.235	11	1	1	7	0	94	**0.003**
INPP4B high	58	16			47	27			27	46			9	60			5	24	45			19	47	7			34	32			20	9	30			12	9	10	26	17		
Any PTEN/PI3K/INPP4B	44	17	95	0.434	37	24	95	0.333	24	36	94	0.305	11	47	89	0.467	2	13	45	94	**0.004**	18	34	8	94	0.561	27	27	84	0.379	20	6	26	78	0.594	14	4	6	18	9	83	0.892
None PTEN/PI3K/INPP4B	27	7			24	10			10	24			4	27			5	13	16			8	21	5			18	12			6	8	12			6	2	5	12	7		
Any PTEN/PI3K/INPP4B/EGFR/HER2	47	33	106	**0.0004**	43	37	106	**0.014**	40	39	105	**0.0004**	19	56	101	**0.0044**	6	17	56	105	0.081	21	47	12	106	0.268	40	33	96	0.559	23	10	30	84	0.319	23	10	9	16	8	92	**<0.0001**
None PTEN/PI3K/INPP4B/EGFR/HER2	25	1			21	5			3	23			0	26			2	12	12			9	15	2			11	12			4	6	11			0	0	2	16	8		

Footnote: Definitions are as in Table 2

*TP53* mutation was significantly positively correlated to PTEN/PI3K pathway alterations when analyzed as the combined pathway hit variable (*P* = 0.0004), and individually to PTEN alterations (*P* = 0.0029) and EGFR overexpression (*P* = 0.0001), but not to *HER2*-amplification, *PIK3CA* mutations, or *INPP4B* loss (*P* = 0.0576, *P* = 0.9663, and *P* = 0.2921, respectively) (Table 3). Of note, 3 of 4 *PTEN* mutants were also *TP53* mutated. Consistent with the literature (see [33] for a review), *TP53* mutations were significantly more common in ER-negative tumors compared to ER-positive tumors (62 % vs. 28 %, *P* = 0.0008); were prevalent in CK5/14^+^ basal-like tumors (61 %; *P* = 0.0341); and the mutational rate increased with grade (0 % mutated in grade 1, 28 % in grade 2, 47 % mutated in grade 3; *P* = 0.0031). No significant associations to patient outcome were found for the tumor group clusters, nor when testing individual or aggregated pathway lesions. This is likely due to the fact that there was a high rate of loss to follow-up (median follow-up 2.5 years, range 0 to 12 years).

Importantly, we found loss of PTEN to be common among IHC-BLBC (61 % vs. 16 % in non-BLBC; *P* < 0.0001; Table 3). Moreover, as expected EGFR overexpression was also significantly associated to the IHC-BLBC subtype (79 % vs. 12 %; *P* < 0.0001). When utilizing the combined pathway hit variable, all IHC-BLBC (100 %) and all GEX-BLBC tumors (100 %) had ≥1 PTEN/PI3K pathway-activating lesion compared to 68 % and 62 % of non-basal-likes (*P* = 0.0044 and *P* = 0.0005).

The significant association between PTEN protein loss and BLBC status was confirmed in the independent series of consecutive breast cancer cases from the Netherlands Cancer Institute (NKI). Corroborating our findings in the Columbia cohort, CK5/14 was a good surrogate for BLBC status in the NKI series with 22 of 25 (88 %) CK5/14-positive cases belonging to the GEX-BLBC subtype (*P* < 0.0001, *n* = 220). Furthermore, PTEN protein loss occurred significantly more often within the BLBC subtype whether defined by microarray subtyping (23/46; 50 % of GEX-BLBC had PTEN-loss; *P* = 0.0003, *n* = 245) or when defined by CK5/14-positivity (15/25; 60 % of IHC-BLBC had PTEN-loss; *P* < 0.0001, *n* = 218). To note, stathmin protein levels were again significantly higher in tumors with PTEN protein loss (*P* = 0.0019, *n* = 237) and in tumors expressing the PTEN-loss gene expression signature (*P* < 0.0001, *n* = 239).

### Combined inhibition of PI3K and EGFR synergistically inhibits BLBC cell growth in vitro

**Fig. 2:**
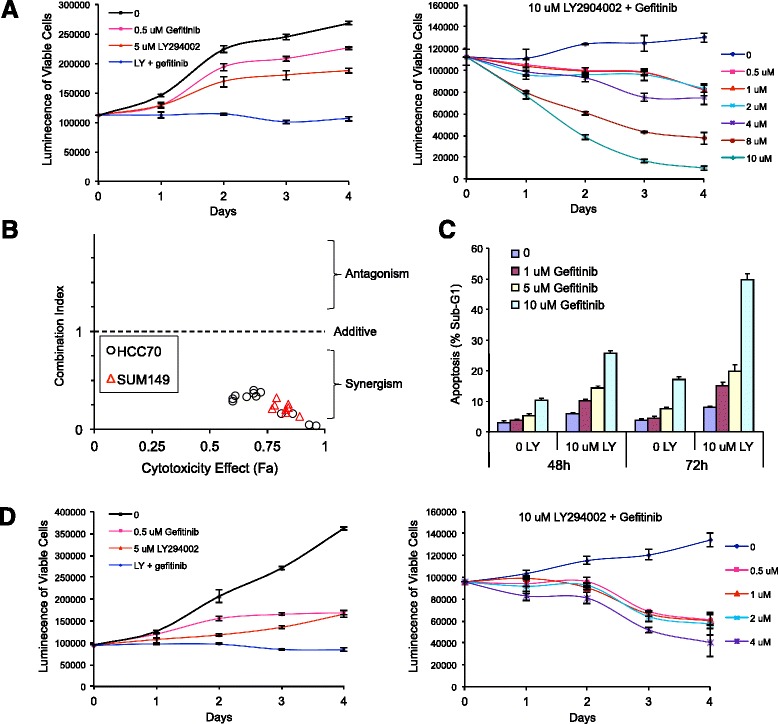
Combined inhibition of PI3K and EGFR synergistically inhibits BLBC cell growth via induction of apoptosis. The growth of the basal-like HCC70 (**a**) or SUM149 (**d**) cells was assessed after 4 days of treatment with single agent or combination treatment with LY294002 and gefitinib at the indicated concentrations. The results are shown as luminescence of viable cells. **b** Combination index plot for 5 or 10 μM LY294002 combined with 0.5, 1, 2, 4, 8, or 10 μM gefitinib (HCC70) or 0.5, 1, 2, 4 μM gefitinib (SUM149). **c** Dose-dependent induction of apoptosis in HCC70 cells by gefitinib and LY294002

Based on these results, we hypothesized that monotherapy against either the PI3K or EGFR pathway nodes in BLBC may be ineffective due to resistance mediated by the alternative uninhibited signaling route, and therefore combination therapy could synergistically overcome this resistance mechanism. We first tested this in vitro using HCC70 BC cells, a relevant model which has high EGFR expression, *PTEN* mutation, and displays the BLBC phenotype by gene expression profiling [30]. When cells were treated from 0.5 μM to 10 μM concentration, the small molecule EGFR inhibitor gefitinib was marginally growth inhibiting at low concentrations and cytostatic at high concentrations (Additional file 1: Figure S1a). However, when combined with 5 μM or 10 μM of the PI3K pathway inhibitor LY294002, dose-dependent synergistic growth inhibition was seen with a combination index of <0.4 at all dose combinations (Fig. 2a-b). The effect of the combination treatment was accompanied by a marked induction of apoptosis (Fig. 2c). Similar synergistic effects for gefitinib plus LY294002 were seen in a second BLBC model cell line, SUM149, which also overexpresses EGFR and is mutant for *PTEN* (Fig. 2c-d; Additional file 1: Figure S1b).

### Combined inhibition of PI3K and EGFR effectively suppresses PTEN-deficient and EGFR-overexpressing BLBC tumor growth in vivo

**Fig. 3:**
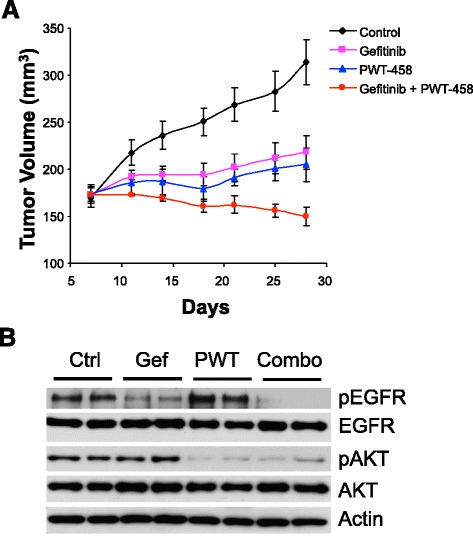
Combination of PI3K and EGFR inhibitors markedly suppresses PTEN-deficient and EGFR-overexpressing BLBC tumor growth in vivo. **a** Mice with established MDA-MB-468 xenograft tumors were treated with PWT-458 (100 mg/kg 5 times/week), gefitinib (150 mg/kg five times/week), combination of both drugs, or vehicle control, and tumor size was measured by caliper two times per week. The results are presented as the mean tumor volume ± S.E.M. (*n* = 5 mice/group). **b** Representative tumors from mice in (**a**) were lysed 5 h after the final treatment with the indicated drugs. Tumor lysates were subjected to Western blot analysis for the indicated proteins

The synergistic anti-proliferative and apoptotic effects of the combination of PI3K pathway and EGFR inhibitors in vitro suggest that targeting both PI3K and EGFR pathways may be a rational strategy for the treatment of BLBC. To explore the feasibility of this therapeutic strategy, we tested the safety and efficacy of inhibiting PI3K and EGFR in vivo using a mouse xenograft model with the BLBC cell line MDA-MB-468 which has DNA amplification and overexpression of EGFR as well as PTEN loss [18, 30, 43]. Similar to the findings observed in HCC70 and SUM149 BCLB cells (Fig. 2 and Additional file 1: Figure S1), we have previously shown that MDA-MB-468 cells also respond poorly to inhibition with LY294002 or gefitinib alone but are very sensitive to combination of these PI3K and EGFR inhibitors for a synergistic induction of apoptosis [18]. Here, we utilized the PI3K-inhibitor, pegylated-17-hydroxywortmannin (PWT-458), which is completely miscible in feed water and relatively stable in plasma [44]. Similar to our in vitro results, administration of PWT-458 or gefitinib alone slowed growth of tumors, but they still grew significantly (Fig. 3a). In contrast, treatment with both drugs profoundly suppressed the growth of the tumor xenografts and caused tumor regression. In addition, chronic administration of both PWT-458 at 100 mg/kg and gefitinib at 150 mg/kg for 5 consecutive days each week for 3 weeks was well tolerated with no weight loss in the animals (Additional file 2: Figure S2). Analysis of xenograft tumors by western blot indicated that gefitinib markedly inhibited EGFR phosphorylation but had no effect on AKT phosphorylation (Fig. 3b). By contrast, PWT-458 effectively caused reduction in AKT phosphorylation but had no effect on EGFR phosphorylation. However, gefitinib in combination with PWT-458 was associated with a profound decrease in the phosphorylation levels of both EGFR and AKT (Fig. 3b). These data highlight the effectiveness of concomitant inhibition of PI3K and EGFR in BLBC.

## Discussion

We show in this study that PTEN/PI3K pathway alterations occur in more than half of an unselected population of 116 human breast carcinomas. Specifically, nearly all ER-negative breast tumors have one or more PTEN/PI3K pathway alterations in stark contrast to the rate in ER-positives. This result may have important clinical implications, as, with the exception of trastuzumab (Herceptin) for *HER2*-amplified BC, there are presently no targeted therapeutic options for patients with ER-negative disease. Our results suggest that therapies that specifically attack the PI3K pathway could be effective in this subtype of BC.

In addition to the previously reported high rate of *TP53* mutations among *HER2*-amplified [33] and EGFR-overexpressing [45] BC, we found *TP53* mutations to be significantly associated to PTEN abrogation. This is highly relevant in the context of recent work which demonstrated in human cells that oncogenic PI3K pathway activation via targeted *PTEN* disruption or *PIK3CA* mutation resulted in stabilization of p53 levels and induction of p53-mediated cellular senescence [46]. It was concluded from this in vitro data that loss of *PTEN* or mutation of *PIK3CA* could elicit selective pressure on tumors to inactivate *TP53* [46]. Our result supports this hypothesis in human breast tumors with respect to PTEN and p53, and further corroborates the cooperative nature between PTEN lesions and p53 inactivation that has also been observed in mouse models [47]. Moreover, our results are consistent with the models proposed relating the p53 stress response pathway to the PTEN/PI3K growth pathway [48]. Of 25 BCs with abrogation of PTEN and with *TP53* mutational data, we found 16 cases (64 %) with coincident mutation of *TP53*, including 3 cases with deleterious mutations to both genes. Therefore, our results demonstrate that the PTEN and p53 tumor suppressors are frequently inactivated in the same individual breast tumors.

Interestingly, *PIK3CA* mutations were significantly associated with lower tumor grade, lower S-phase fraction, and reduced Ki67 staining. This may indicate that, in vivo, *PIK3CA* mutation is a less potent driver of cell proliferation than, for example, PTEN alteration. The association of *PIK3CA* mutations to markers of favorable prognosis and to good outcome are consistent with other recent reports [49–51]. We previously reported that *PIK3CA* mutations were positively associated to ER, lymph node status, and *HER2* status [2]. Our present study finds similar trends, however the associations were not significant may be due to the fact that the present study analyzed fewer cases compared to our prior report. We found reduced expression of *INPP4B* in ER-negative breast tumors as well as occurring most frequently in BLBC, consistent with prior reports [6, 9, 10].

All cases in our identified cluster A had one or more PTEN/PI3K pathway activating lesions, and this cluster closely corresponds to the basal-like subtype of BC as defined by either CK5/14 positive staining or by GEX-based intrinsic molecular subtyping. Our tumor marker profiling informed a therapeutic strategy for co-targeting the EGFR and PTEN/PI3K nodes. This hypothesis was tested in vitro and in vivo and we show that cluster A-type BLBC tumors are sensitive to the combination therapy with the EGFR and PTEN/PI3K pathway inhibitors. Moreover, our data suggests that it could be clinically feasible to identify such patients using IHC panels or by gene expression profiling. It is interesting how PTEN/PI3K pathway lesions and our identified tumor clusters may relate to the other molecular subtypes. For example, our cluster C2/C3 corresponded most closely to the luminal A subtype, with prominent hormone receptor expression, a low *TP53* mutation rate, and reduced proliferation. The C2 cluster also contained no *PIK3CA* mutants, thus one would hypothesize that cases with this profile would respond best to selective ER modulators such as tamoxifen or to aromatase inhibitors [52–54]. Our clusters B and C4 correspond closely to the HER2 subtype, and most of these tumors have intact PTEN and are *PIK3CA* wild-type, making them ideal for traztuzumab-containing regimens [15, 17]. Subcluster C5 is ER^+^ with *TP53* mutation and has an overlap with the luminal B class. Given that PI3K pathway activation has been implicated in tamoxifen resistance [52–54] and *TP53* mutations in resistance to polychemotherapy with cyclophosphamide, methotrexate, and 5-fluorouracil [55], we would hypothesize that tumors with a pathway profile like cluster C5 may require more intensive regimens than standard treatment. These hypotheses require further evaluation in appropriate models and patient material.

We found that BLBC tumors frequently have coincident EGFR-overexpression and loss of PTEN, which may implicate their selective advantage for maintaining BLBC transformation and contributing to the aggressive BLBC characteristics. We have previously found that in MDA-MB-468 BLBC cells with coexistent EGFR-overexpression and PTEN loss, inhibition of EGFR with gefitinib has no effect on PI3K/AKT signaling but effectively suppresses MAPK signaling [18]. Thus, PTEN-deficient MDA-MB-468 cells proliferate and survive in an EGFR-independent manner. However, restoration of PTEN or inhibition of PI3K with LY294002 causes these cells to depend on EGFR/MAPK signaling pathway for survival, whereas combined inhibition of both PI3K/AKT and EGFR/MAPK signaling pathways synergistically induces apoptosis. Mechanistically, we have identified the BAD protein as a switch that integrates the antiapoptotic effects of the PI3K/AKT and EGFR/MAPK pathways [18]. Each pathway is responsible for phosphorylation of BAD at a distinct site. Genetic induction of PTEN expression in combination of with the EGFR inhibitor gefitinib synergistically suppresses PTEN-deficient and EGFR-overexpressing BLBC tumor growth and causes tumor regression [18]. The current study demonstrates effective synergy in vitro and in vivo using combination of pharmacological inhibitors targeting both EGFR and PI3K for the treatment of BLBC with concurrent PTEN loss and EGFR overexpression. We cannot rule out that off-target effects of LY294002 may have contributed to the synergistic effects observed when combined with gefitinib. Moreover, PMT-458 is a pan-PI3K inhibitor. To further delineate the signaling, these studies can be improved upon by the use of more specific inhibitors. Nevertheless, together our studies provide a heuristic model for understanding pathway interactions and suggest that combination therapy with EGFR/MAPK and PI3K/AKT pathway inhibitors is a rational strategy for BLBC treatment and deserves further pre-clinical/clinical testing. Our results parallel the situation seen in glioblastoma multiforme in which overexpression or amplification of EGFR and its variant, EGFRvIII, is commonly associated with *PTEN* deletion or mutation. This suggests that the coexistence of EGFR activation with PTEN loss may be a common paradigm in cancer that could potentially be exploited. This hypothesis should be extended beyond breast and brain cancers. For example, EGFR and PTEN appear to be involved in the pathogenesis of lung adenocarcinoma, however the prevalence of both lesions is not well studied. If loss of PTEN is frequent among EGFR-positive cancer, then this may be a contributing factor to the significant resistance to EGFR inhibitors seen in the clinic [16] and further stresses the potential importance of combination therapies against both EGFR/MAPK and PTEN/PI3K/AKT signaling pathways. Interestingly, inhibition of AKT signaling appears to activate some RTKs via de-repression of RTK expression and increased RTK phosphorylation, including that for EGFR in some tumor models [56]. Together, our data suggest that a promising strategy may be to co-target RTKs such as EGFR when targeting the PI3K/AKT pathway, and vice versa.

In summary, we have revealed novel relationships between PTEN/PI3K pathway lesions and of these lesions to the BC subtypes. Furthermore, we show that aberrant PTEN/PI3K signaling is closely correlated to mutation of *TP53*. Our biomarker and therapy results have important and obvious clinical implications for stratifying patients and designing clinical trials that target the PTEN/PI3K pathway as well as the EGFR/HER2 receptors and MAPK pathway. Finally, the fact that the majority of BCs have oncogenic PTEN/PI3K pathway lesions associated with the worst prognosis highlights the enormous need and potential benefit for targeting this pathway using recently developed PI3K and AKT inhibitors.

## Conclusions

We have performed a comprehensive molecular characterization of unselected breast cancers and show a high rate of PTEN/PI3K pathway-related alterations in ER-negative breast cancer, an in particular show that basal-like breast cancers often display concomitant overexpression of EGFR and loss PTEN, the negative regulator of PI3K. We evaluated a combination therapy co-targeting EGFR and PI3K in models of basal-like breast cancer in vitro and demonstrate synergistic anti-cancer effects that shrinks tumors in vivo, with greater efficacy than either monotherapy. Our results support the clinical evaluation of combining EGFR/MAPK and PI3K/AKT pathway inhibitors for the treatment of BLBC.

## Abbreviations

BC, breast cancer; BLBC, basal-like breast cancer; CI, combination index; CK5/14, cytokeratins 5 and 14; DAB, diaminobenzidine; ER, estrogen receptor; FFPE, formalin-fixed paraffin-embedded; IHC, immunohistochemistry; PgR, progesterone receptor; PI3K, phosphoinositide 3-kinase; PIP2, phosphatidylinositol 3,4-bisphosphate; TMA, tissue microarray

## Additional files

Additional file 1: Figure S1. HCC70 (A) or SUM149 (B) cells were treated with single-agent gefitinib (left) or combination treatment with 5 μM LY294002 plus gefitinib at the concentrations indicated and cell proliferation measured over 4 days. (PDF 217 kb) Additional file 2: Figure S2. Chronic treatment with gefitinib and PWT-458, alone or in combination, does not cause eight loss in mice. Mice bearing MDA-MB-468 xenograft tumors were treated with PWT-458 (100 mg/kg five imes/week), gefitinib (150 mg/kg five times/week), combination of both drugs, or vehicle control. The mouse body weight was measured in control and treated groups using a weighing scale. The results represent the mean body weight ± S.E.M. (n = 5 mice per group). (PDF 880 kb)

## References

[1] Rakha E, Reis-Filho JS (2009). Basal-like breast carcinoma: from expression profiling to routine practice. Arch Pathol Lab Med.

[2] Saal LH, Holm K, Maurer M, Memeo L, Su T, Wang X, Yu JS, Malmstrom PO, Mansukhani M, Enoksson J (2005). PIK3CA mutations correlate with hormone receptors, node metastasis, and ERBB2, and are mutually exclusive with PTEN loss in human breast carcinoma. Cancer Res.

[3] Li SY, Rong M, Grieu F, Iacopetta B (2006). PIK3CA mutations in breast cancer are associated with poor outcome. Breast Cancer Res Treat.

[4] Stemke-Hale K, Gonzalez-Angulo AM, Lluch A, Neve RM, Kuo WL, Davies M, Carey M, Hu Z, Guan Y, Sahin A (2008). An integrative genomic and proteomic analysis of PIK3CA, PTEN, and AKT mutations in breast cancer. Cancer Res.

[5] Miron A, Varadi M, Carrasco D, Li H, Luongo L, Kim HJ, Park SY, Cho EY, Lewis G, Kehoe S (2010). PIK3CA mutations in in situ and invasive breast carcinomas. Cancer Res.

[6] The Cancer Genome Atlas (2012). Comprehensive molecular portraits of human breast tumours. Nature.

[7] Saal LH, Gruvberger-Saal SK, Persson C, Lovgren K, Jumppanen M, Staaf J, Jonsson G, Pires MM, Maurer M, Holm K (2008). Recurrent gross mutations of the PTEN tumor suppressor gene in breast cancers with deficient DSB repair. Nature Genet.

[8] Martins FC, De S, Almendro V, Gonen M, Park SY, Blum JL, Herlihy W, Ethington G, Schnitt SJ, Tung N (2012). Evolutionary pathways in BRCA1-associated breast tumors. Cancer Discov.

[9] Fedele CG, Ooms LM, Ho M, Vieusseux J, O'Toole SA, Millar EK, Lopez-Knowles E, Sriratana A, Gurung R, Baglietto L (2010). Inositol polyphosphate 4-phosphatase II regulates PI3K/Akt signaling and is lost in human basal-like breast cancers. Proc Natl Acad Sci U S A.

[10] Gewinner C, Wang ZC, Richardson A, Teruya-Feldstein J, Etemadmoghadam D, Bowtell D, Barretina J, Lin WM, Rameh L, Salmena L (2009). Evidence that inositol polyphosphate 4-phosphatase type II is a tumor suppressor that inhibits PI3K signaling. Cancer Cell.

[11] Agoulnik IU, Hodgson MC, Bowden WA, Ittmann MM (2011). INPP4B: the new kid on the PI3K block. Oncotarget.

[12] Bjorge JD, Chan TO, Antczak M, Kung HJ, Fujita DJ (1990). Activated type I phosphatidylinositol kinase is associated with the epidermal growth factor (EGF) receptor following EGF stimulation. Proc Natl Acad Sci U S A.

[13] Peles E, Lamprecht R, Ben-Levy R, Tzahar E, Yarden Y (1992). Regulated coupling of the Neu receptor to phosphatidylinositol 3'-kinase and its release by oncogenic activation. J Biol Chem.

[14] Eccles SA (2011). The epidermal growth factor receptor/Erb-B/HER family in normal and malignant breast biology. Int J Dev Biol.

[15] Nagata Y, Lan KH, Zhou X, Tan M, Esteva FJ, Sahin AA, Klos KS, Li P, Monia BP, Nguyen NT (2004). PTEN activation contributes to tumor inhibition by trastuzumab, and loss of PTEN predicts trastuzumab resistance in patients. Cancer Cell.

[16] Mellinghoff IK, Wang MY, Vivanco I, Haas-Kogan DA, Zhu S, Dia EQ, Lu KV, Yoshimoto K, Huang JH, Chute DJ (2005). Molecular determinants of the response of glioblastomas to EGFR kinase inhibitors. N Engl J Med.

[17] Berns K, Horlings HM, Hennessy BT, Madiredjo M, Hijmans EM, Beelen K, Linn SC, Gonzalez-Angulo AM, Stemke-Hale K, Hauptmann M (2007). A functional genetic approach identifies the PI3K pathway as a major determinant of trastuzumab resistance in breast cancer. Cancer Cell.

[18] She QB, Solit DB, Ye Q, O'Reilly KE, Lobo J, Rosen N (2005). The BAD protein integrates survival signaling by EGFR/MAPK and PI3K/Akt kinase pathways in PTEN-deficient tumor cells. Cancer Cell.

[19] Bianco R, Shin I, Ritter CA, Yakes FM, Basso A, Rosen N, Tsurutani J, Dennis PA, Mills GB, Arteaga CL (2003). Loss of PTEN/MMAC1/TEP in EGF receptor-expressing tumor cells counteracts the antitumor action of EGFR tyrosine kinase inhibitors. Oncogene.

[20] She QB, Solit D, Basso A, Moasser MM (2003). Resistance to gefitinib in PTEN-null HER-overexpressing tumor cells can be overcome through restoration of PTEN function or pharmacologic modulation of constitutive phosphatidylinositol 3'-kinase/Akt pathway signaling. Clin Cancer Res.

[21] van de Vijver MJ, He YD, van't Veer LJ, Dai H, Hart AA, Voskuil DW, Schreiber GJ, Peterse JL, Roberts C, Marton MJ (2002). A gene-expression signature as a predictor of survival in breast cancer. N Engl J Med.

[22] Nuyten DS, Hastie T, Chi JT, Chang HY, van de Vijver MJ (2008). Combining biological gene expression signatures in predicting outcome in breast cancer: An alternative to supervised classification. Eur J Cancer.

[23] Chen Y, van de Vijver MJ, Hibshoosh H, Parsons R, Saal LH (2016). PTEN and NEDD4 in Human Breast Carcinoma. Pathol Oncol Res.

[24] Laakso M, Loman N, Borg A, Isola J (2005). Cytokeratin 5/14-positive breast cancer: true basal phenotype confined to BRCA1 tumors. Mod Pathol.

[25] Saal LH, Johansson P, Holm K, Gruvberger-Saal SK, She QB, Maurer M, Koujak S, Ferrando AA, Malmstrom P, Memeo L (2007). Poor prognosis in carcinoma is associated with a gene expression signature of aberrant PTEN tumor suppressor pathway activity. Proc Natl Acad Sci U S A.

[26] Tanner M, Gancberg D, Di Leo A, Larsimont D, Rouas G, Piccart MJ, Isola J (2000). Chromogenic in situ hybridization: a practical alternative for fluorescence in situ hybridization to detect HER-2/neu oncogene amplification in archival breast cancer samples. Am J Pathol.

[27] Troein C, Vallon-Christersson J, Saal LH (2006). An introduction to BioArray Software Environment. Methods Enzymol.

[28] Hu Z, Fan C, Oh DS, Marron JS, He X, Qaqish BF, Livasy C, Carey LA, Reynolds E, Dressler L (2006). The molecular portraits of breast tumors are conserved across microarray platforms. BMC Genomics.

[29] Chou TC, Talalay P (1984). Quantitative analysis of dose-effect relationships: the combined effects of multiple drugs or enzyme inhibitors. Adv Enzyme Regul.

[30] She QB, Chandarlapaty S, Ye Q, Lobo J, Haskell KM, Leander KR, DeFeo-Jones D, Huber HE, Rosen N (2008). Breast tumor cells with PI3K mutation or HER2 amplification are selectively addicted to Akt signaling. PLoS One.

[31] de Hoon MJ, Imoto S, Nolan J, Miyano S (2004). Open source clustering software. Bioinformatics.

[32] Saldanha AJ (2004). Java Treeview--extensible visualization of microarray data. Bioinformatics.

[33] Borresen-Dale AL (2003). TP53 and breast cancer. Hum Mutat.

[34] Carey LA, Perou CM, Livasy CA, Dressler LG, Cowan D, Conway K, Karaca G, Troester MA, Tse CK, Edmiston S (2006). Race, breast cancer subtypes, and survival in the Carolina Breast Cancer Study. JAMA.

[35] Bachman KE, Argani P, Samuels Y, Silliman N, Ptak J, Szabo S, Konishi H, Karakas B, Blair BG, Lin C (2004). The PIK3CA Gene is Mutated with High Frequency in Human Breast Cancers. Cancer Biol Ther.

[36] Bose S, Crane A, Hibshoosh H, Mansukhani M, Sandweis L, Parsons R (2002). Reduced expression of PTEN correlates with breast cancer progression. Hum Pathol.

[37] Campbell IG, Russell SE, Choong DY, Montgomery KG, Ciavarella ML, Hooi CS, Cristiano BE, Pearson RB, Phillips WA (2004). Mutation of the PIK3CA gene in ovarian and breast cancer. Cancer Res.

[38] Depowski PL, Rosenthal SI, Ross JS (2001). Loss of expression of the PTEN gene protein product is associated with poor outcome in breast cancer. Mod Pathol.

[39] Perren A, Weng LP, Boag AH, Ziebold U, Thakore K, Dahia PL, Komminoth P, Lees JA, Mulligan LM, Mutter GL (1999). Immunohistochemical evidence of loss of PTEN expression in primary ductal adenocarcinomas of the breast. Am J Pathol.

[40] Abd El-Rehim DM, Pinder SE, Paish CE, Bell JA, Rampaul RS, Blamey RW, Robertson JF, Nicholson RI, Ellis IO (2004). Expression and co-expression of the members of the epidermal growth factor receptor (EGFR) family in invasive breast carcinoma. Br J Cancer.

[41] Nielsen TO, Hsu FD, Jensen K, Cheang M, Karaca G, Hu Z, Hernandez-Boussard T, Livasy C, Cowan D, Dressler L (2004). Immunohistochemical and clinical characterization of the basal-like subtype of invasive breast carcinoma. Clin Cancer Res.

[42] Biganzoli E, Coradini D, Ambrogi F, Garibaldi JM, Lisboa P, Soria D, Green AR, Pedriali M, Piantelli M, Querzoli P (2011). p53 status identifies two subgroups of triple-negative breast cancers with distinct biological features. Jpn J Clin Oncol.

[43] Li J, Yen C, Liaw D, Podsypanina K, Bose S, Wang SI, Puc J, Miliaresis C, Rodgers L, McCombie R (1997). PTEN, a putative protein tyrosine phosphatase gene mutated in human brain, breast, and prostate cancer. Science.

[44] Yu K, Lucas J, Zhu T, Zask A, Gaydos C, Toral-Barza L, Gu J, Li F, Chaudhary I, Cai P (2005). PWT-458, a novel pegylated-17-hydroxywortmannin, inhibits phosphatidylinositol 3-kinase signaling and suppresses growth of solid tumors. Cancer Biol Ther.

[45] Fox SB, Harris AL (1997). The epidermal growth factor receptor in breast cancer. J Mammary Gland Biol Neoplasia.

[46] Kim JS, Lee C, Bonifant CL, Ressom H, Waldman T (2007). Activation of p53-dependent growth suppression in human cells by mutations in PTEN or PIK3CA. Mol Cell Biol.

[47] Chen Z, Trotman LC, Shaffer D, Lin HK, Dotan ZA, Niki M, Koutcher JA, Scher HI, Ludwig T, Gerald W (2005). Crucial role of p53-dependent cellular senescence in suppression of Pten-deficient tumorigenesis. Nature.

[48] Levine AJ, Feng Z, Mak TW, You H, Jin S (2006). Coordination and communication between the p53 and IGF-1-AKT-TOR signal transduction pathways. Genes Dev.

[49] Loi S, Haibe-Kains B, Majjaj S, Lallemand F, Durbecq V, Larsimont D, Gonzalez-Angulo AM, Pusztai L, Symmans WF, Bardelli A (2010). PIK3CA mutations associated with gene signature of low mTORC1 signaling and better outcomes in estrogen receptor-positive breast cancer. Proc Natl Acad Sci U S A.

[50] Di Cosimo S, Baselga J (2009). Phosphoinositide 3-kinase mutations in breast cancer: a "good" activating mutation?. Clin Cancer Res.

[51] Janku F, Wheler JJ, Naing A, Falchook GS, Hong DS, Stepanek VM, Fu S, Piha-Paul SA, Lee JJ, Luthra R (2013). PIK3CA mutation H1047R is associated with response to PI3K/AKT/mTOR signaling pathway inhibitors in early-phase clinical trials. Cancer Res.

[52] Campbell RA, Bhat-Nakshatri P, Patel NM, Constantinidou D, Ali S, Nakshatri H (2001). Phosphatidylinositol 3-kinase/AKT-mediated activation of estrogen receptor alpha: a new model for anti-estrogen resistance. J Biol Chem.

[53] Clark AS, West K, Streicher S, Dennis PA (2002). Constitutive and inducible Akt activity promotes resistance to chemotherapy, trastuzumab, or tamoxifen in breast cancer cells. Mol Cancer Ther.

[54] Kirkegaard T, Witton CJ, McGlynn LM, Tovey SM, Dunne B, Lyon A, Bartlett JM (2005). AKT activation predicts outcome in breast cancer patients treated with tamoxifen. J Pathol.

[55] Andersson J, Larsson L, Klaar S, Holmberg L, Nilsson J, Inganas M, Carlsson G, Ohd J, Rudenstam CM, Gustavsson B (2005). Worse survival for TP53 (p53)-mutated breast cancer patients receiving adjuvant CMF. Ann Oncol.

[56] Chandarlapaty S, Sawai A, Scaltriti M, Rodrik-Outmezguine V, Grbovic-Huezo O, Serra V, Majumder PK, Baselga J, Rosen N (2011). AKT inhibition relieves feedback suppression of receptor tyrosine kinase expression and activity. Cancer Cell.

