# Supplementary material for: Integrated molecular pathway analysis informs a synergistic combination therapy targeting PTEN/PI3K and EGFR pathways for basal-like breast cancer
Source: BMC Cancer. 2016 Aug 2;16:587. doi: 10.1186/s12885-016-2609-2 (PMC4971667; doi:10.1186/s12885-016-2609-2)
Supplement: Additional file 1: Figure S1. — HCC70 (A) or SUM149 (B) cells were treated with single-agent gefitinib (left) or combination treatment with 5 μM LY294002 plus gefitinib at the concentrations indicated and cell proliferation measured over 4 days. (PDF 217 kb) [file 12885_2016_2609_MOESM1_ESM.pdf]

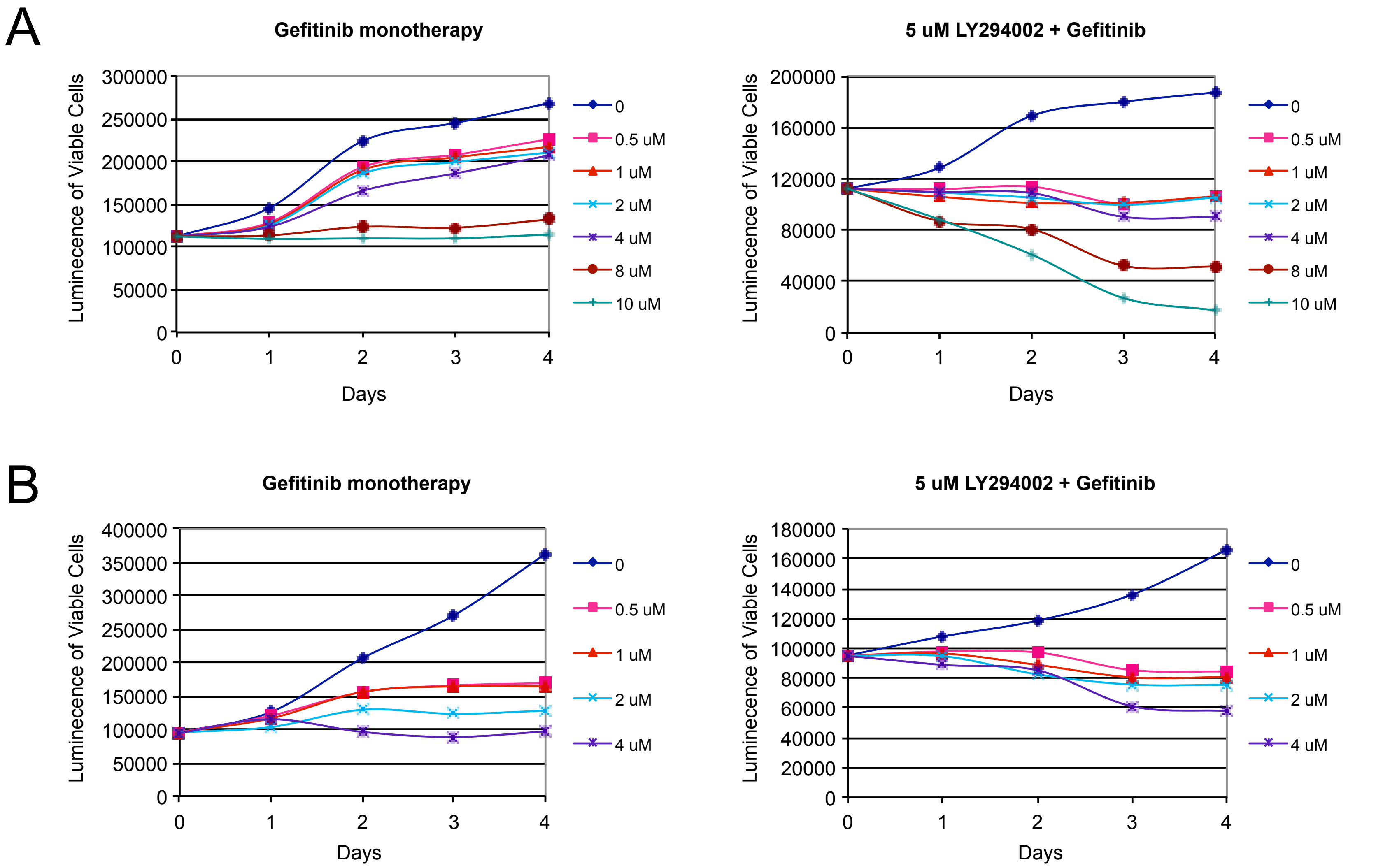

Supplementary Figure 1 – HCC70 (**A**) or SUM149 (**B**) cells were treated with single-agent gefitinib (left) or combination treatment with 5 uM LY294002 plus gefitinib at the concentrations indicated and cell proliferation measured over 4 days.
