# Supplementary material for: Integrated molecular pathway analysis informs a synergistic combination therapy targeting PTEN/PI3K and EGFR pathways for basal-like breast cancer
Source: BMC Cancer. 2016 Aug 2;16:587. doi: 10.1186/s12885-016-2609-2 (PMC4971667; doi:10.1186/s12885-016-2609-2)
Supplement: Additional file 2: Figure S2. — Chronic treatment with gefitinib and PWT-458, alone or in combination, does not cause eight loss in mice. Mice bearing MDA-MB-468 xenograft tumors were treated with PWT-458 (100 mg/kg five imes/week), gefitinib (150 mg/kg five times/week), combination of both drugs, or vehicle control. The mouse body weight was measured in control and treated groups using a weighing scale. The results represent the mean body weight ± S.E.M. (n = 5 mice per group). (PDF 880 kb) [file 12885_2016_2609_MOESM2_ESM.pdf]

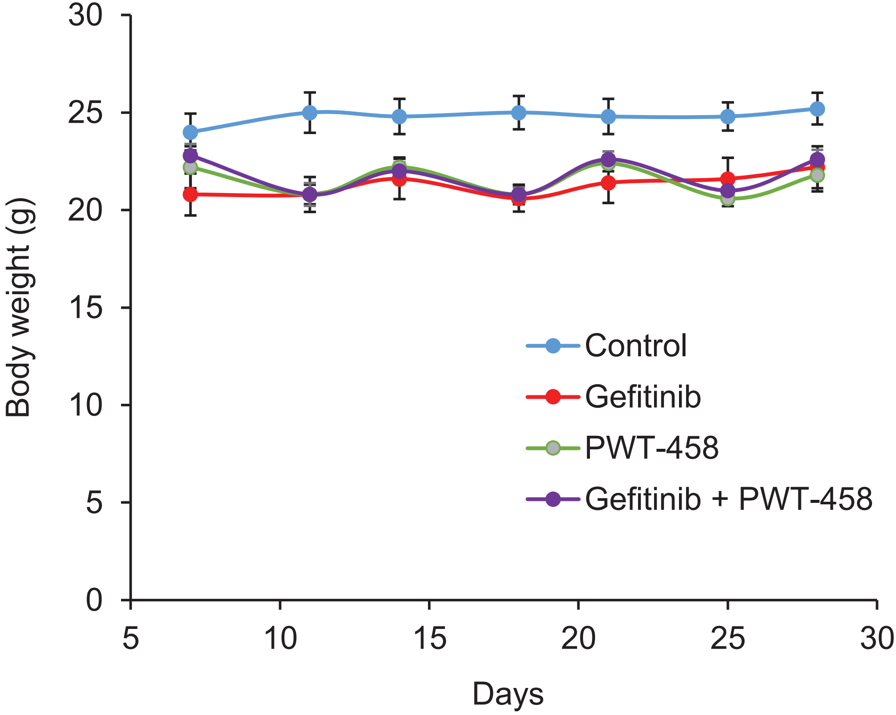

Supplementary Figure 2 - Chronic treatment with gefitinib and PWT-458, alone or in combination, does not cause weight loss in mice. Mice bearing MDA-MB-468 xenograft tumors were treated with PWT-458 (100 mg/kg five times/week), gefitinib (150 mg/kg five times/week), combination of both drugs, or vehicle control. The mouse body weight was measured in control and treated groups using a weighing scale. The results represent the mean body weight ± S.E.M. (n = 5 mice per group).
